# Effectiveness of short-term heat acclimation on intermittent exercise in thermoneutral and hot environments

**DOI:** 10.1186/2046-7648-4-S1-A120

**Published:** 2015-09-14

**Authors:** Fiona Nation, Matt Birkett, Damien Gleadall-Siddall, Rachel Burke, Christopher Towlson, James Bray, Grant Abt, Andrew Garrett

**Affiliations:** 1 Department of Sport, Health and Exercise Science, Exercise Health and Human Performance Research Group, University of Hull, UK

## Introduction

It is well-established that repetition of heat stress exposure has been shown to facilitate adaptations to the heat but these protocols have tended to be of a fixed work intensity, continuous exercise, long-term in duration (>7 days) and use hydration. Secondly, there is limited information on the potential use of heat acclimation as a training method for human performance in thermoneutral conditions [1]. Therefore, the aims of this study were to investigate the effectiveness of short-term heat acclimation (STHA) for 5 days, using the controlled-hyperthermia technique with dehydration, on intermittent exercise in thermoneutral and hot environments.

## Methods

Ten, healthy, active, moderately-trained males (Mean (SD); Age 25.6 (8.9) yrs; Height 180.7 (5.6) cm; Body Mass 83.2 (10.8) kg; **$\overset{\cdot }{\text{V}}{\text{O}}_{2\text{max}}$**45.3 (6.5) mL.kg^-1^.min^-1 ^and resting cardiac output 6.3 (1.8) L.min^-1^), participated in a STHA programme. This consisted of 90 minutes dehydration heat acclimation (no fluid intake) for 5 consecutive days (39.5 °C; 60% rh), using the controlled-hyperthermia technique (~rectal temperature [T_re_] 38.5 °C) [2]. The pre and post testing Exercise Stress Test (EST) consisted of 45 minutes of intermittent exercise (nine phases of 5 minutes). Including resting, walking, moderate and high intensity running) on a motorised, h/p Cosmos treadmill; and nine 6 second (s) maximal sprints on a Watt Bike, as a repeated, maximal sprint performance test. The EST was followed by a running, incremental test to exhaustion. This EST intermittent protocol was adapted from exercise intensities of professional football players [3]. The EST was repeated in controlled conditions (C; 11 °C 45% rh); pre and post STHA in thermoneutral (TN; 11 °C 45% rh) and hot environments (H; 35 °C 45% rh). Data analysis was by paired t-test and reported as the mean differences with 95% confidence intervals (95%CI).

## Results

Post STHA, in the H trial at 45-min there was a decrease in T_re _by -0.2 °C (95%CI: -0.40 to 0.00 °C; p = 0.03), cardiac frequency (-3: -5 to -1 b.min^-1^; p = 0.01) and RPE (-2: -3 to -1 units; p = 0.01). Mean average power (MavP) increased in sprints 7 (111: 25 to 197 W; p = 0.01) and 9 (240: 9 and 489 W; p = 0.04). The increase in Sprint 8 (87: -8 to 182 W; p = 0.06) and time to exhaustion (208: -24 to 439 s; p = 0.06) were close to significance. In the TN trial, increased MavP for sprint 9 (67: 2 to 131 W; p = 0.04) and time to exhaustion (43: 1 to 85 s; p = 0.04) were reported. There was limited change in the C conditions across all nine sprints (p > 0.05) and for time to exhaustion (-31: -72 to 11 s; p = 0.12).

## Discussion

Short-term heat acclimation (5 days) with dehydration, using the controlled-hyperthermia technique, is effective for physiological adaptations during intermittent exercise in a hot environment. Furthermore, it has resulted in an increase in intermittent and endurance exercise performance in hot and thermoneutral conditions.

## Conclusion

Short-term heat acclimation is effective for intermittent exercise in the heat. It may be a useful training method for human performance in thermoneutral conditions.
